# Online learning fuzzy echo state network with applications on redundant manipulators

**DOI:** 10.3389/fnbot.2024.1431034

**Published:** 2024-07-15

**Authors:** Yanqiu Li, Huan Liu, Hailong Gao

**Affiliations:** ^1^School of Data Science and Artificial Intelligence, Jilin Engineering Normal University, Changchun, China; ^2^Department of Basic Sciences, Jilin Jianzhu University, Changchun, China

**Keywords:** echo state network (ESN), fuzzy inference system (FIS), online learning, redundant manipulators, optimization

## Abstract

Redundant manipulators are universally employed to save manpower and improve work efficiency in numerous areas. Nevertheless, the redundancy makes the inverse kinematics of manipulators hard to address, thus increasing the difficulty in instructing manipulators to perform a given task. To deal with this problem, an online learning fuzzy echo state network (OLFESN) is proposed in the first place, which is based upon an online learning echo state network and the Takagi–Sugeno–Kang fuzzy inference system (FIS). Then, an OLFESN-based control scheme is devised to implement the efficient control of redundant manipulators. Furthermore, simulations and experiments on redundant manipulators, covering UR5 and Franka Emika Panda manipulators, are carried out to verify the effectiveness of the proposed control scheme.

## 1 Introduction

To improve production efficiency and set themselves free from manpower, robots have come into being and undergone expeditious and substantial progress, with plentiful and triumphant applications in numerous areas (Sun et al., 2023b; Liu et al., 2024). Therefore, redundant manipulators that possess more degrees of freedom (DOFs) than non-redundant ones to fulfill a specific task stand out and have been subject to in-depth and comprehensive investigations (Liao et al., 2016; Liu et al., 2023). More precisely, by virtue of the additional DOFs, they are capable of executing some secondary tasks while performing the primary task, such as obstacle avoidance, optimizing joint torques, and enhancing operability (Jin et al., 2017a; Sun et al., 2022a). For that reason, research on the mechanisms and applications of redundant manipulators is in full swing. However, it is worth mentioning that the additional DOFs result in troubles and challenges for controlling manipulators efficiently and precisely (Zhang et al., 2019; Zhao et al., 2020). Therefore, it imports the demand to devise and construct a potent control scheme of redundant manipulators (Jin et al., 2017b; Liao et al., 2022).

With a sophisticated and ingenious nervous system, humans are capable of performing a variety of complicated and intractable missions by learning from recent experiences, which is the most prominent difference and superiority compared with other creatures (Wang et al., 2016; Liao et al., 2024b). Therefore, this has opened up a new avenue for the control of manipulators. That is, manipulators can accomplish the assigned task with high efficiency by simulating the learning ability of humans. Taking the neural network (NN) (Su et al., 2023a; Wei and Jin, 2024) and fuzzy inference system (FIS) (Vargas et al., 2024) into account, both of them attempt to simulate the thinking and decision-making processes of humans in a certain way. Therefore, they have garnered the attention of researchers, and a lot of effort has been put into integrating them with manipulator control systems to improve the completion of the task and meet the requirements of different scenarios. For instance, Yoo and Ham (2000) present adaptive control schemes for manipulators, in which the parameter uncertainty is handled via the FIS. Afterward, aiming at the tracking control of the end-effector for manipulators, an FIS-based controller is designed by Yilmaz et al. (2022), in which the centers and widths of the membership functions are adjusted adaptively, thus promoting the learning power of the controller. Recently, Yilmaz et al. (2023) devised an FIS-based output-feedback controller for the joint space tracking of manipulators, in which the demands for joint velocity and knowledge of manipulators are eliminated.

In recent times, a surge of research has come into view in the realm of the echo state network (ESN), a sort of recurrent neural network (RNN), which overcomes certain problems hindering the investigations and applications of RNNs, such as gradient vanishing and gradient exploding (Rodan and Tino, 2011; Chen et al., 2023). The core of ESN lies in the reservoir, which is a large, sparse network in charge of capturing the dynamic behavior of input information. Particularly in the ESN, both input and reservoir weights are generated at random, and one needs to put effort into obtaining the output weights by figuring out the weighted sum of outputs (Lukoševičius, 2012). Considering another network, the extreme learning machine (ELM) (Huang et al., 2006) is a feedforward network with a hidden layer. Weights and biases for the hidden layer are appointed randomly, while the training of the network focuses on determining output weights through the least squares method. Therefore, from the perspective of this point, the ELM, ESN, and FIS share a certain similarity, and thus, a great deal of work has been carried out that builds and verifies the bridges between them (Sun et al., 2007; Ribeiro et al., 2020). By integrating these networks and taking advantage of their strengths, some extraordinary work is presented and utilized in various domains to address different issues. Concentrating on function approximation and classification problems, a fuzzy ELM with the capacity for online learning was devised by Rong et al. (2009). Compared with other existing mechanisms it presents remarkable superiority with decent accuracy and reduced training time. Motivated by this, aiming at efficient control of redundant manipulators, this study proposes an online learning fuzzy ESN (OLFESN). To be more specific, the proposed OLFESN is designed, based on an online learning strategy for ESN, to erect an efficient control scheme for redundant manipulators, while the FIS is also incorporated to improve the accuracy and efficiency of the proposed network. Then, a corresponding control scheme for redundant manipulators is constructed. The rest of this study is organized as follows: Section 2 makes known some preliminary steps to lay the foundation for this study. Then, the OLFESN is proposed, based on which the control scheme for redundant manipulators is devised in Section 3. In Section 4, simulations and experiments are carried out to investigate the feasibility and effectiveness of the proposed control scheme. In the end, Section 5 concludes this study.

## 2 Preliminaries

In this section, the forward kinematics of redundant manipulators, the Takagi–Sugeno–Kang (TSK) fuzzy system, and ESN are briefly reviewed, which are the bases of the proposed OLFESN.

### 2.1 Forward kinematics of redundant manipulators

The forward kinematics equation that depicts the non-linear transformation of redundant manipulators from the joint angle **q** ∈ ℝ^*a*^ to the Cartesian position **r** ∈ ℝ^*b*^ with *a* > *b* can be depicted as


(1) $$\begin{array}{l} ϒ\left(\text{q}\right)=\text{r}, \end{array}$$

where ϒ(•) signifies the non-linear mapping function, which depends upon the structural properties of redundant manipulators (Sun et al., 2022b; Zhang et al., 2022). Where after, evaluating the derivative of Equation (1) in terms of time contributes to


(2) $$\begin{array}{l} J\left(\text{q}\right)\dot{\text{q}}=\dot{\text{r}}, \end{array}$$

in which *J*(**q**) = ∂ϒ(**q**)/∂**q** ∈ ℝ^*b* × *a*^ denotes the Jacobian matrix; $\dot{\text{q}}\;$ denotes the angular velocity; $\dot{\text{r}}$ denotes the velocity of the end-effector (Yan et al., 2024). Heretofore, the non-linear transformation (Equation 1) is converted to the affine system (Equation 2) with the convenience of gaining the redundancy solution of redundant manipulators (Sun et al., 2023a).

### 2.2 Takagi–Sugeno–Kang fuzzy system

In the TSK fuzzy system with given input $\alpha =\left[\alpha_{1};\alpha_{2};\cdots ;\alpha_{m}\right]\in {\mathbb{R}}^{m}$, the *k*-th rule can be depicted as Kerk et al. (2021) and Zhang et al. (2023):


(3) $$\begin{array}{r} Rule\;k:IF\;\alpha_{1}is\;A_{1k},\;\alpha_{2}\;is\;A_{2k},\cdots ,\alpha_{m}is\;A_{mk},\\ THEN\;\chi_{k}=\beta_{0k}+\beta_{1k}\alpha_{1}+\beta_{2k}\alpha_{2}+\cdots +\beta_{mk}\alpha_{m}, \end{array}$$

where $k=1,2,\cdots ,\tilde{k}$ is the index of the fuzzy rule with $\tilde{k}\;$ being the number of fuzzy rules; *A*_*mk*_ denotes the fuzzy subset of the *m*-th element of input α in the *k*-th rule; χ_*k*_ signifies the output of the *k*-th rule; $\beta_{\tilde{m}k}\left(\tilde{m}=0,1,\cdots ,m\right)$ is the consequent coefficient of the *k*-th rule. Considering the *m*-th element of input in the *k*-th rule, the degree to which it matches the fuzzy subset *A*_*mk*_ is measured by its membership function *ζ*_*A*_*mk*__(α_*m*_), which can be any bounded non-constant piecewise continuous function (Rezaee and Zarandi, 2010). Let ⊗ denote the fuzzy conjunction operation, and then the firing strength (if part) of the *k*-th rule is defined as


(4) $$\begin{array}{l} O_{k}\left(\alpha ,{\text{p}}_{\text{k}}\right)=\zeta_{A_{1k}}\left(\alpha_{1},p_{1,k}\right)⊗\zeta_{A_{2k}}\left(\alpha_{2},p_{2,k}\right)⊗\cdots ⊗\zeta_{A_{mk}}\left(\alpha_{m},p_{m,k}\right), \end{array}$$

where **p**_*k*_ is the parameter of membership function ζ(•) in the *k*-th rule. Normalizing (Equation 4), there is


(5) $$\begin{array}{l} \text{Ψ}\left(\alpha ,{\text{p}}_{\text{k}}\right)=\frac{O_{k}\left(\alpha ,{\text{p}}_{\text{k}}\right)}{\overset{\tilde{k}}{\underset{k=1}{\sum }}O_{k}\left(\alpha ,{\text{p}}_{\text{k}}\right)}. \end{array}$$

Ultimately, for the input α, the output of the TSK fuzzy model can be obtained as


(6) $$\tilde{y}=\frac{\sum_{k=1}^{\tilde{k}}\theta_{k}O_{k}(\alpha ,p_{k})}{\sum_{k=1}^{\tilde{k}}O_{k}(\alpha ,p_{k})}=\sum_{k=1}^{\tilde{k}}\theta_{k}\Psi (\alpha ,p_{k}),$$

with *θ*_*k*_ = (*θ*_*k*1_, *θ*_*k*2_, ⋯ , *θ*_*km*_).

### 2.3 Echo state network

The ESN is composed of an input layer, a reservoir, and an output layer, which enjoy *l, r*, and *o* neurons, respectively (Calandra et al., 2021). For a complete network, the input layer, reservoir, and output layer re connected by input weights $W_{in}\in {\mathbb{R}}^{r\times l}$ and output weights $W_{out}\in {\mathbb{R}}^{o\times r}$, respectively, while the internal neurons of the reservoir are connected to each other by dint of $W_{res}\in {\mathbb{R}}^{r\times r}$ (Chen et al., 2024a). In particular, the spectral radius of *W*_*res*_ needs to be < 1 to capture the echo state property. At the time of step *i*, designate input and reservoir states as ${\text{x}}_{i}=\left[x_{1};x_{2};\cdots ;x_{l}\right]\in {\mathbb{R}}^{l}$ and $\iota_{i}=\left[\iota_{1};\iota_{2};\cdots ;\iota_{r}\right]\in {\mathbb{R}}^{r}$, respectively. The reservoir is updated through


(7) $$\begin{array}{l} \iota_{i}=f\left(W_{in}x_{i}+W_{res}\iota_{\left(\text{i}-1\right)}\right), \end{array}$$

and the output of the network is


(8) $$\begin{array}{l} y_{i}=g\left(W_{out}\iota_{i}\right), \end{array}$$

with ${\text{y}}_{i}=\left[y_{1};y_{2};\cdots ;y_{o}\right]\in {\mathbb{R}}^{o}$. Furthermore, for working out the output weights, keep track of reservoir state and outputs in matrices $\Lambda =\left[\iota_{1},\iota_{2},\cdots ,\iota_{\tilde{i}}\right]\in {\mathbb{R}}^{r\times \tilde{i}}$ and $Y=\left[y_{1},y_{2},\cdots ,y_{\tilde{i}}\right]\in {\mathbb{R}}^{o\times \tilde{i}}$, respectively, during training, where ĩ denotes the number of training samples. Where after, by solving


(9) $$\begin{array}{l} \underset{W_{\text{out}}}{\min}:||Y-W_{out}\Lambda |{|}_{2,}^{2} \end{array}$$

the output weights are obtained


(10) $$\begin{array}{l} W_{out}=Y\Lambda^{T}{\left(\Lambda \Lambda^{T}\right)}^{-1} \end{array}$$

where the superscripts ^T^ and ^−1^ represent transpose and inversion operations of a matrix, respectively (Su et al., 2023b; Liao et al., 2024a).

## 3 Online learning fuzzy echo state network

Stimulated by the commonalities between ESN and FIS, OLFESN is proposed in this section. Then, an OLFESN-based control scheme for redundant manipulators is devised.

### 3.1 OLFESN

Considering (Equation 4), the firing strength (if any) in the TSK fuzzy system involves multiple fuzzy conjunction operations, providing sufficient computing power for thoroughly exploring and utilizing input information. Furthermore, each rule is normalized to ensure that different rules have a comparable contribution to the system. Similarly, in the ESN, it is the reservoir that is responsible for implementing the above function, by which the low-dimensional input is mapped to a high-dimensional dynamic space. In addition, the outputs of different reservoirs are adjusted to the same extent with the aid of the activation function *f*(•), which plays the same role as Equation (5). Therefore, the reservoir is adopted to reveal the firing strength normalized in the proposed OLESN. Specifically, the OLESN with $\tilde{k}$ reservoirs is established as follows:

Given training samples $ℶ={\left\{\left(x_{i},y_{i}\right)\right\}}_{i=1}^{\tilde{i}}$, the state of the *k*-th reservoir is updated via


(11) $$\iota_{ki}=f_{k}(W_{in}x_{i}+W_{res}\iota_{k(i-1)}),\;i=1,2,\cdots ,\tilde{i},$$

where *f*_*k*_(•) denotes the activation function of the *k*-th reservoir, and ĩ is the number of training samples. Collect all states of the *k*-th reservoir in Ξ_*k*_ = [**ι**_*k*1_, **ι**_*k*2_, ⋯ , **ι**_*kĩ*_, and then integrate all $\tilde{k}$ reservoirs elicited


(12) $$\begin{array}{l} \Lambda =\Xi_{1}\Xi_{2}\cdots \Xi_{\tilde{k}}. \end{array}$$

Thus, the output of the fuzzy ESN (FESN) can be formulated as


(13) $$\begin{array}{l} Y=W_{out}\Lambda , \end{array}$$

with *Y* = [**y**_1_, **y**_2_, ⋯ , **y**_ĩ_]. Similarly to Equation (10), output weights are obtained via


(14) $$\begin{array}{l} W_{out}=Y\Lambda^{T}{\left(\Lambda \Lambda^{T}\right)}^{-1}. \end{array}$$

At this point, the derivation of FESN is complete. Therewith, taking into account the need for online learning, the OLFESN is proposed, which incorporates the FESN and the online learning strategy for ESN. To be more specific, when data shows up constantly, the OLFESN is summarized as follows:

### 3.2 Initialization phase

a. Given the initial training samples ${ℶ}_{0}={\left\{\left({\text{x}}_{i},{\text{y}}_{i}\right)\right\}}_{i=1}^{{\tilde{i}}_{0}}$, update and transcribe the state of all $\tilde{k}$ reservoirs using Equation 11.

b. Taking advantage of Equation 12, figure out the initial state matrix Λ_0_ for FESN.

c. Compute the initial output weights $W_{out}^{0}=T_{0}\Lambda_{0}^{T}Y_{0}$ with $T_{0}={\left(\Lambda_{0}^{T}\Lambda_{0}\right)}^{-1}$ and *Y*_0_ = [**y**_1_, **y**_2_, ⋯ , **y**_ĩ_0__].

d. Let *p* = 0.

### 3.3 Sequential learning phase

a. With the new sample set


$$\begin{array}{l} {ℶ}_{p+1}={\left\{\left({\text{x}}_{i},{\text{y}}_{i}\right)\right\}}_{i=\left(\overset{p}{\underset{j=0}{\sum }}{\tilde{i}}_{j}\right)+1}^{\overset{p+1}{\underset{j=0}{\sum }}{\tilde{i}}_{j}}, \end{array}$$

solve problem


(15) $$\begin{array}{l} ||W_{out}^{p+1}\left[\Lambda_{p,\;}\Lambda_{p+1\;}\right]-\left[Y_{p,\;}Y_{p+1\;}\right]|{|}_{2}^{2}, \end{array}$$

where ĩ_*p*+1_ signifies the count of samples in the (*p*+1)-th set; Λ_*p*+1_is the corresponding reservoir state, obtained by Equations 11, 12; $Y_{p+1\;}=\left[{\text{y}}_{\left(\overset{p}{\underset{j=0}{\sum }}{\tilde{i}}_{j}\right)+1},\cdots ,{\text{y}}_{\left(\overset{p+1}{\underset{j=0}{\sum }}{\tilde{i}}_{j}\right)+\;1}\right]$.

b. Let ${\text{Ψ}}_{p}=H_{p}^{-1}$ with $H_{P}=\left[\Lambda_{p,\;}\Lambda_{p+1\;}\right]{\left[\Lambda_{p,\;}\Lambda_{p+1}\right]}^{T}$.

c. Update output weights


(16) $$\begin{array}{r} {\text{Ψ}}_{p+1}={\text{Ψ}}_{p}-{\text{Ψ}}_{p}\Lambda_{p+1\;}{\left(I+\Lambda_{p+1}^{T}{\text{Ψ}}_{p}\Lambda_{p+1\;}\right)}^{-1}\Lambda_{p+\;1}^{T}{\text{Ψ}}_{p},\\ W_{out}^{p+1}=W_{out}^{p}+\left(Y_{p+1\;}-W_{out}^{p}\Lambda_{p+1\;}\right)\;\Lambda_{p+1}^{T}{\text{Ψ}}_{p+1}. \end{array}$$

d. Let *p* = *p* + 1. (Back to step 2).

*Remark 1*: For the case that the new samples come out one by one, with the aid of the Sherman-Morrison formula (Chen et al., 2024b), Equation 16 is further simplified as


(17) $$\begin{array}{r} {\text{Ψ}}_{p+1}={\text{Ψ}}_{p}-\frac{{\text{Ψ}}_{p}\iota_{p+1}\iota_{p+1}^{T}{\text{Ψ}}_{p}}{1+\iota_{p+\;1}^{T}{\text{Ψ}}_{p}\iota_{k1}},\\ W_{out}^{p+1}=W_{out}^{p}+\left({\text{y}}_{p+1\;}-W_{out}^{p}\iota_{p+1\;}\right)\;\iota_{p+1}^{T}{\text{Ψ}}_{p+1}. \end{array}$$

### 3.4 OLFESN-based control scheme

In this section, an OLFESN-based control scheme for redundant manipulators is developed for performing the given missions. At moment *t*, define *θ*_*a*_ (*t*) and Δ*θ*_*a*_ (*t*) as the actual joint angle and actual joint angle increment, respectively. Meanwhile, the actual and desired positions of the end-effector are denoted by **ζ**_*a*_ (*t*) and **ζ**_*d*_ (*t*), respectively. Correspondingly, at moment *t*+1, the desired position increment for the end-effector is expressed as Δ**ζ**(*t*+1) = **ζ**_*d*_(*t*)−**ζ**_*a*_(*t*). Incorporate *θ*_*a*_(*t*), Δ*θ*_*a*_(*t*), and Δ**ζ**(*t* + 1), which is the input of the OLFESN and denoted by **x**(*t*) for the convenience of subsequent expressions. Then, applying Equations 11–13, we gain the joint angle increment Δ*θ*_*a*_(*t* + 1) for the next moment, i.e., the output of OLFESN. Hence, the control signal for the next moment is acquired, i.e., *θ*_*a*_(*t*+1) = *θ*_*a*_(*t*) + Δ*θ*_*a*_(*t* + 1 ).

Note that, in the OLFESN, there is a premise that sample (**x**(*t*), **y**(*t*)) is accessible all the time. However, for the proposed scheme, the desired joint angle increment Δ*θ*_*d*_(*t*+1), i.e., **y**(*t*), is unrevealed in reality. In addition, taking into account output weights *W*_*out*_, it ought to be updated in real-time to generate the control signal. An accepted wisdom is making use of the teaching signal to update output weights *W*_*out*_. More specifically, the error **ϵ**(*t*+1) between the desired joint angle increment Δ*θ*_*d*_(*t*+1) and the actual one Δ*θ*_*a*_(*t*+1) plays a part in the teaching signal in the proposed scheme.

Informed by Equation (2), the transformation between joint angle increment Δ*θ*(*t*) and position increment Δ**ζ**(*t*) of the end-effector is devised as


(18) $$\begin{array}{l} J\left(t\right)\Delta \theta \left(t\right)=\;\Delta \zeta \left(t\right). \end{array}$$

Then, we have


(19) $$\begin{array}{r} \zeta_{d}\left(t+1\right)-\zeta_{a}\left(t+1\right)=J\left(t+1\right)\left(\theta_{d}\left(t+1\right)-\theta_{a}\left(t+1\right)\right)\\ =J\left(t+1\right)\left(\theta_{a}\left(t\right)-{\Delta \theta }_{d}\left(t+1\right)-\left(\theta_{a}\left(t\right)+{\Delta \theta }_{a}\left(t+1\right)\right)\right)\\ =J\left(t+1\right)\left({\Delta \theta }_{d}\left(t+1\right)-{\Delta \theta }_{a}\left(t+1\right)\right). \end{array}$$

Solving Equation 19, the teaching signal is collected as


(20) $$\begin{array}{l} \epsilon \left(t+1\right)=J^{+}\left(t+1\right)\left(\zeta_{d}\left(t+1\right)-\zeta_{a}\left(t+1\right)\right). \end{array}$$

Until now, the proposed control scheme for redundant manipulators based on the above-mentioned teaching signal and OLFESN has been constructed as


(21) $$\begin{array}{c} \text{Ψ}\left(t+1\right)=\text{Ψ}\left(t\right)-\text{Ψ}\left(t\right)\Lambda \left(t+1\right){\left(I+{\Lambda \left(t+1\right)}^{T}\text{Ψ}\left(t\right)\Lambda \left(t+1\right)\right)}^{-1}\\ {\Lambda \left(t+1\right)}^{T}\text{Ψ}\left(t\right), \end{array}$$

$$\begin{array}{l} W_{out}\left(t+1\right)=W_{out}\left(t\right)+\epsilon \left(t+1\right){\Lambda \left(t+1\right)}^{T}\text{Ψ}\left(t+1\right), \end{array}$$

which is outlined and summarized in Algorithm 1.

**Algorithm 1:**
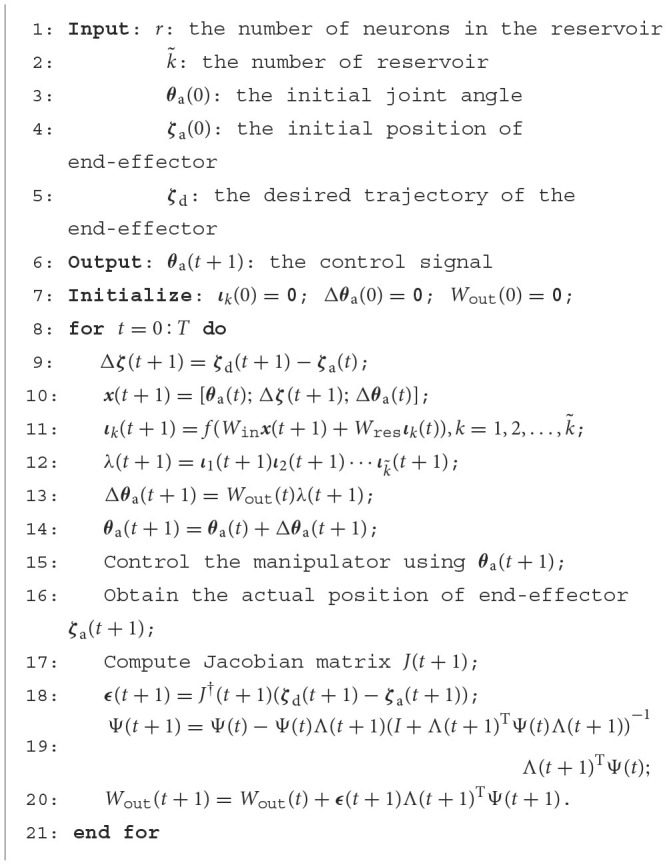
Proposed Control Scheme

## 4 Illustrative examples

In this section, simulations on redundant manipulators are devised and executed, covering a 6-DOF manipulator and a 7-DOF one, to verify the effectiveness and feasibility of the proposed scheme (Equation 21).

### 4.1 UR5

**Figure 1:**
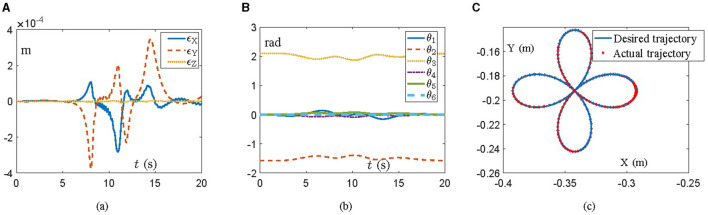
Simulative experiment results on the UR5 manipulator synthesized by the proposed scheme (Equation 21) for tracking a four-leaf clover path. **(A)** Profiles of the tracking error. **(B)** Profiles of the joint angle. **(C)** The desired trajectory and the actual trajectory.

A UR5 manipulator is employed with the aid of the proposed scheme (Equation 21) in this simulation, which possesses 6 DOFs and is explicitly revealed in Zheng et al. (2019) and Chico et al. (2021). The task is to track a four-leaf clover path within 20 s, where the initial angle state is *θ*(0) = [0; −π/2; 2π/3; 0; 0; 0] rad. With regard to OLFESN, the input weights *W*_*in*_ and internal connection weights of reservoir *W*_*res*_ are randomly initialized to [−0.5, 0.5] by using MATLAB's 2022 *rand*(•) function. In addition, we bring in a total of three reservoirs, each with 500 neurons and the hyperbolic tangent function (tanh(•)), while the spectral radius is set to 0.8. Specifically, simulation results are exhibited in Figure 1, where Figure 1A illustrates the position errors of the end-effector during task execution. One can observe that the manipulator, with the aid of the proposed scheme (Equation 21), does the job with flying colors, and the position error of the end-effector is of the order 10^−4^ m. Correspondingly, trails of joint angles and task completion are shown in Figures 1B, C, respectively. Note that, during the task, the joint angles of the manipulator are evolving in a gentle manner, which is capable of reducing the wear between mechanical components to a certain extent, thus elongating the service life of the manipulator. In the end, Figure 1C further indicates that the task of tracking the four-leaf clover path is commendably accomplished by the manipulator, with the actual trajectory synthesized by the proposed scheme (Equation 21) excellently covering the desired one.

### 4.2 Franka Emika panda manipulator

**Figure 2:**
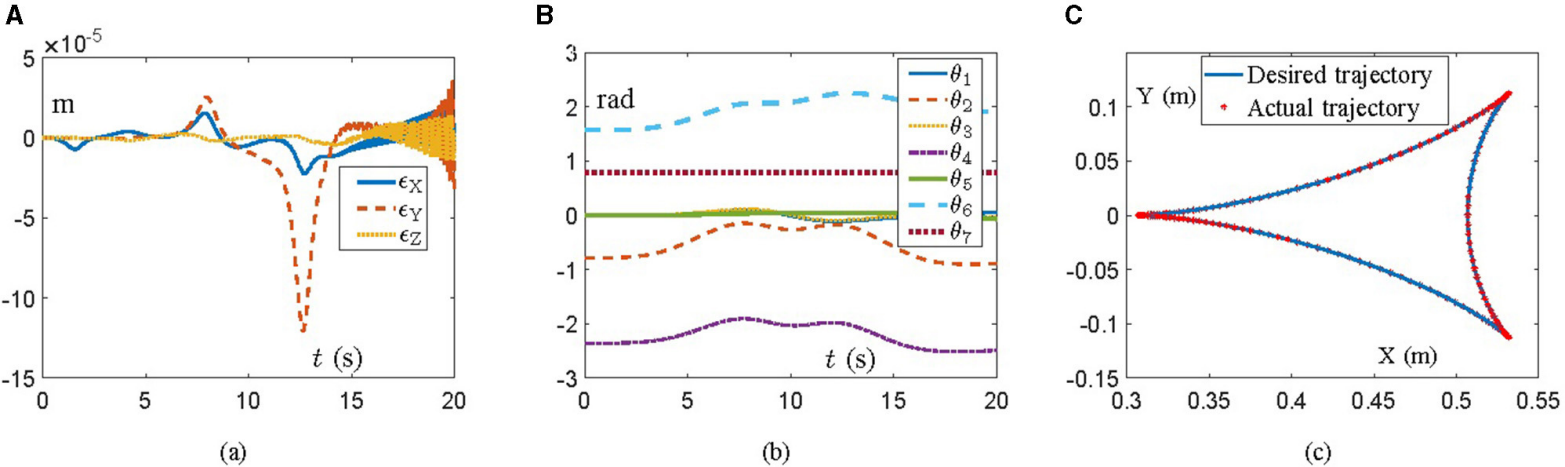
Simulative experiment results on the Frank Emika Panda manipulator synthesized by the proposed scheme (Equation 21) for tracking a tricuspid valve trajectory. **(A)** Profiles of the tracking error. **(B)** Profiles of the joint angle. **(C)** The desired trajectory and the actual trajectory.

**Figure 3:**
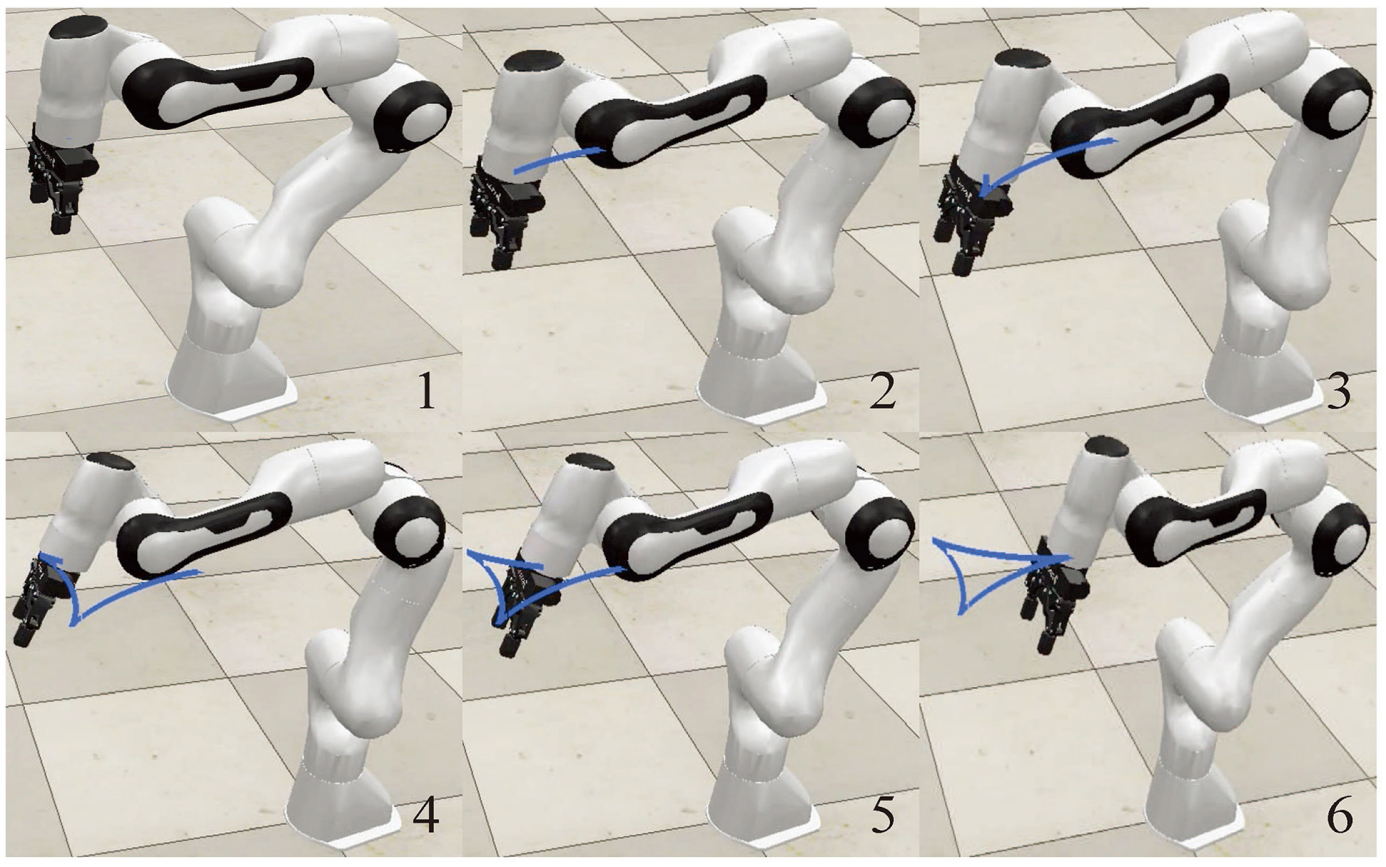
Snapshots of the Franka Emika Panda manipulator simulated on the (V-REP) platform for tracking the tricuspid valve trajectory with the aid of the proposed scheme (Equation 21).

In this part, the simulation of a Franka Emika Panda manipulator is designed and carried out to further verify the effectiveness and feasibility of the proposed scheme (Equation 21). The Franka Emika Panda is a 7-DOF manipulator with structural information covered by Shahid et al. (2020) and Gaz et al. (2019), which is necessary to track a tricuspid valve trajectory within 20 s. The initial angle state is *θ*(0) = [0; −π/4; 0; −3π/4; 0;π/2; π/4] rad, while the other parameters are in line with those in Section 4.1. Figure 2 reveals simulation results, where position errors of the end-effector are exhibited in Figure 2A. Viewing position errors, one can lightly draw the conclusion that the Franka Emika Panda manipulator controlled by the proposed scheme (Equation 21) finishes the given task successfully, with the position error being of the order 10^−5^ m. Then, pay attention to the variation of joint angles and task completion, which are depicted in Figures 2B, C, respectively. All these results indicate the success of the task, which further verifies the feasibility and effectiveness of the proposed scheme (Equation 21) in the field of robot control. Furthermore, the corresponding simulation experiments are executed on the virtual robot experimentation platform (V-REP) to vividly simulate task execution. Snapshots of the Franka Emika Panda manipulator with the aid of the proposed scheme (Equation 21) are displayed in Figure 3, from which we can observe that the Franka Emika Panda manipulator safely and efficiently performs the task of tracking the tricuspid valve trajectory, thus further verifying the reliability of the above simulation results and the practicability of the proposed scheme (Equation 21).

## 5 Conclusion

Based on the online learning strategy for ESN and FIS, an OLFESN has been proposed, in which the new data is allowed to arrive one by one or in blocks. There are no additional restrictions on the size of blocks, thus highly extending the application scenarios of the proposed OLFESN. Subsequently, to cope with the complicated control problem of redundant manipulators, an OLFESN-based control scheme has been constructed from a kinematics point of view. In the end, simulations and experiments on the UR5 and Franka Emika Panda manipulators have been carried out and confirmed the effectiveness and feasibility of the proposed control scheme (Equation 21). Incorporating joint constraints into the proposed scheme (Equation 21) is a future research direction, that is capable of improving the safety and efficiency of task execution.

## Data availability statement

The raw data supporting the conclusions of this article will be made available by the authors, without undue reservation.

## Ethics statement

The manuscript presents research on animals that do not require ethical approval for their study.

## Author contributions

YL: Formal analysis, Funding acquisition, Methodology, Writing – original draft, Writing – review & editing. HL: Data curation, Methodology, Writing – original draft. HG: Data curation, Formal analysis, Writing – original draft.
